# Supplementary material for: The Patchy Distribution of Restriction–Modification System Genes and the Conservation of Orphan Methyltransferases in Halobacteria
Source: Genes (Basel). 2019 Mar 19;10(3):233. doi: 10.3390/genes10030233 (PMC6471742; doi:10.3390/genes10030233)
Supplement: Supplementary file 1 [file genes-10-00233-s001.zip › Suppl_Figures.pdf]

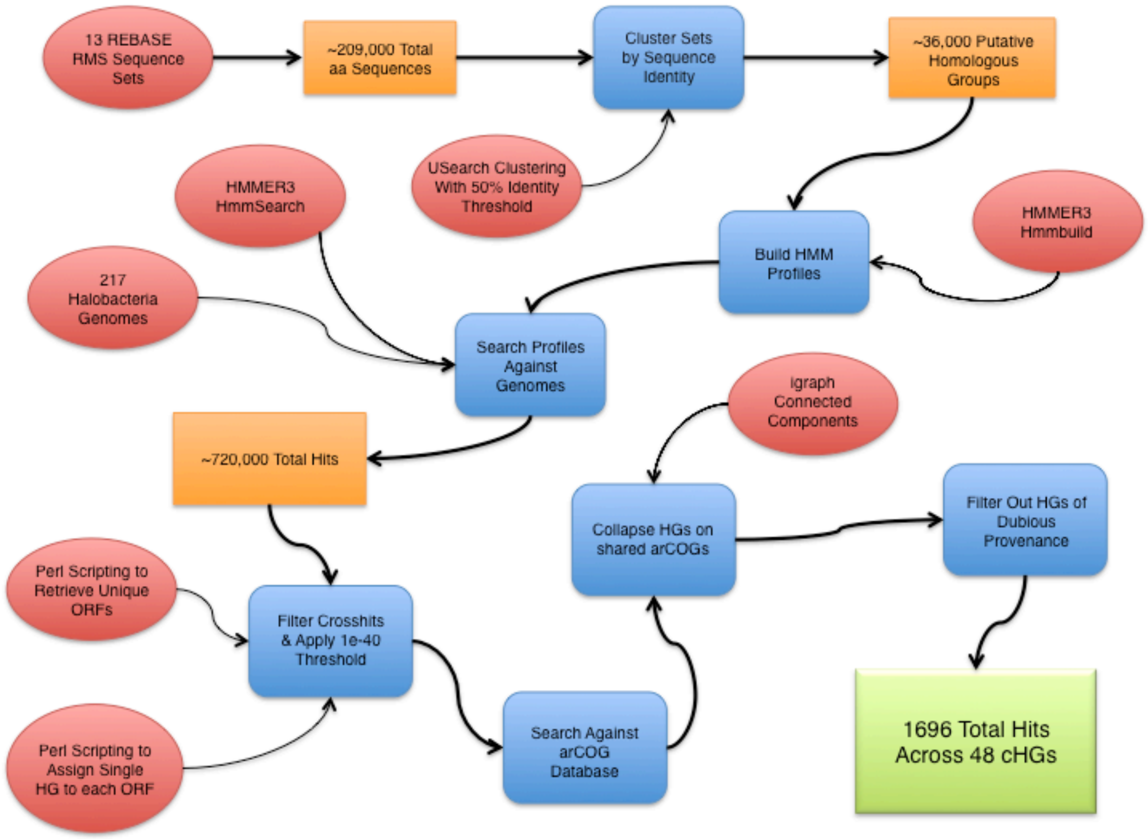

Figure S1. Workflow of RMS-candidate gene search strategy. Red circles represent input data and tools used to operate on data. Blue rectangles represent use and modification of the data during the process. Orange rectangles represent outputs at significant intermediate points in the process.

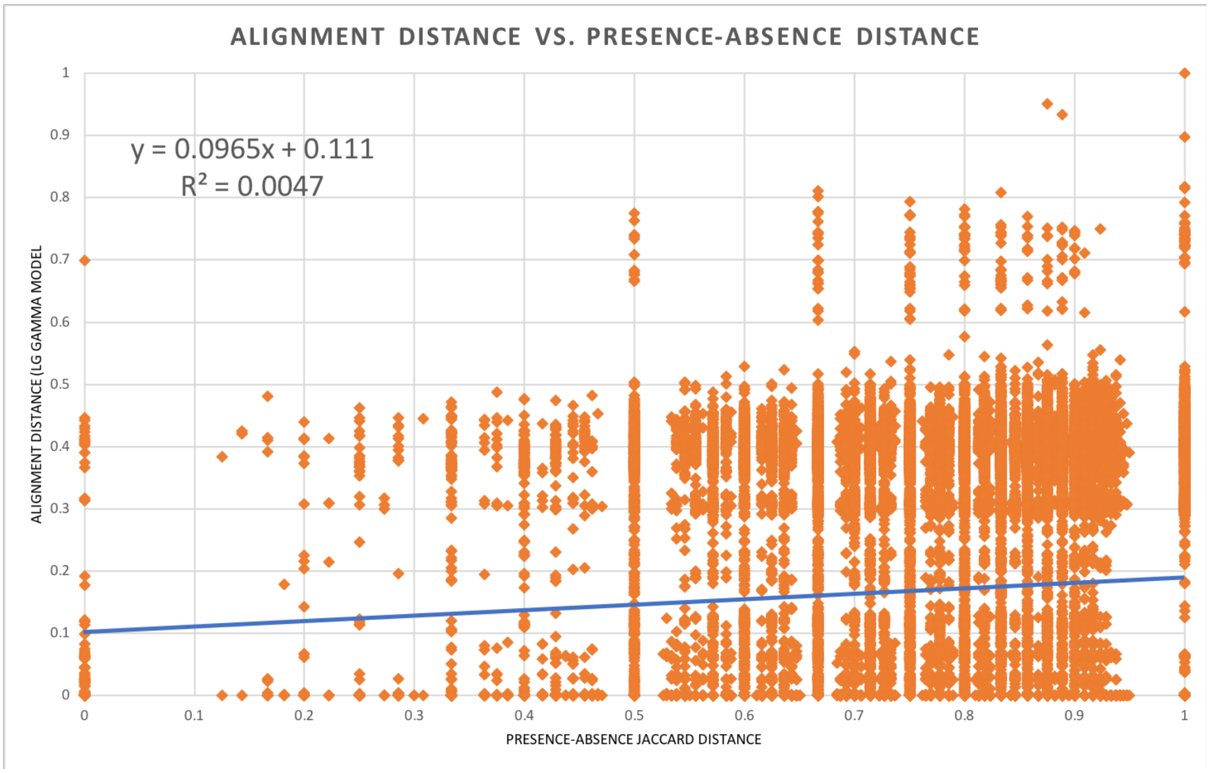

Figure S2. Plot of alignment distance as a function of presence-absence distance. Alignment distance was calculated from an LG+Gamma model and presence-absence using the Jaccard distance. If presence-absence pattern were a function of vertical descent, then the best fit line should broadly follow a line through the origin (0/0). The correlation between the two measures is negligible (r-squared = 0.0047).

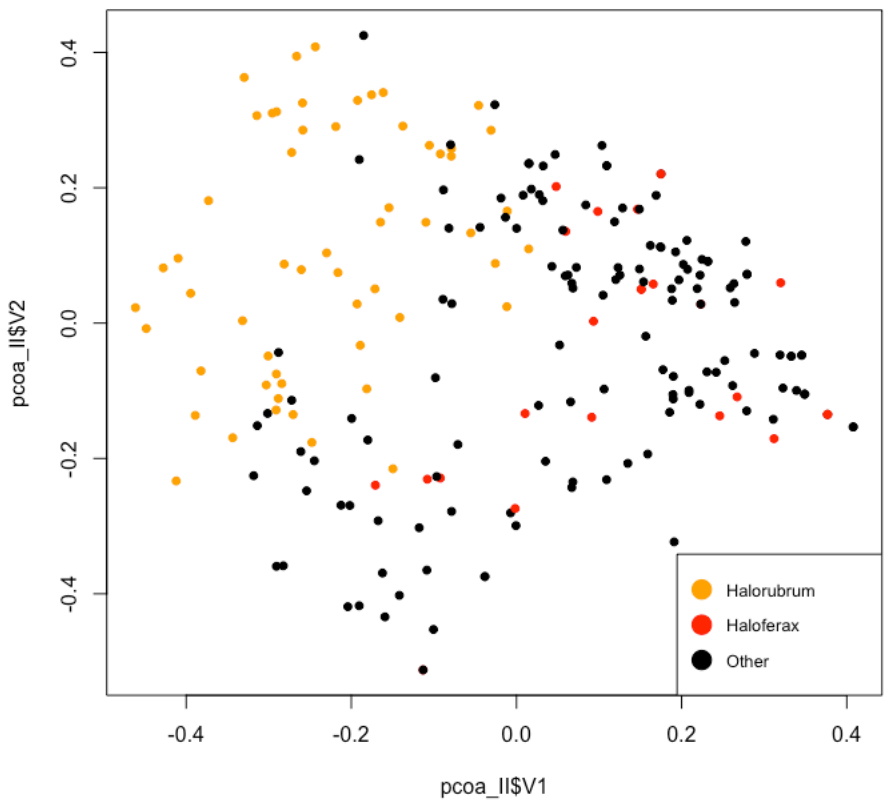

Figure S3. PCoA plot of the distances between the RMS presence-absence profiles of the 217 analyzed Halobacterial genomes. The two best sampled groups, *Halorubrum* (orange) and *Haloferax* (red), are colored separately. The overlap of ranges between the three groups illustrates that few RM system genes are unique to a given group and a large overlap in inventories between groups.

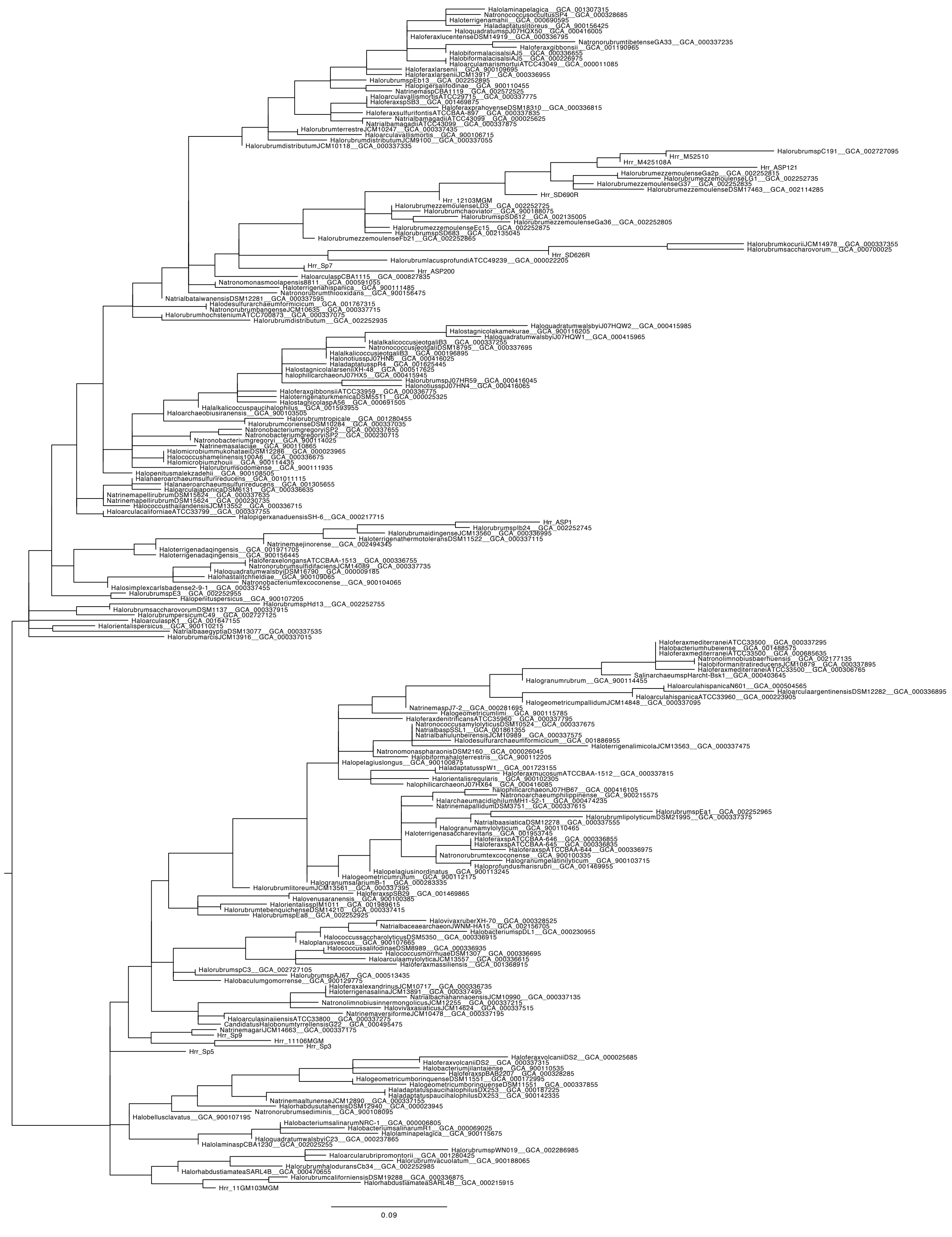
Halolaminapelagica__GCA_001307315
NatronococcusoccultusSP4__GCA_000328685
Haloterrigenamahii__GCA_000690595
Haladaptatuslitoreus__GCA_900156425
HaloquadratumspJ07HQX50__GCA_000416005
HaloferaxlucentenseDSM14919__GCA_000336795
NatronorubrumtibetenseGA33__GCA_000337235
Haloferaxgibbonsii__GCA_001190965
HalobiformalacisalsiAJ5__GCA_000336655
HalobiformalacisalsiAJ5__GCA_000226975
HaloarculamarismortuiATCC43049__GCA_000011085
Haloferaxlarsenii__GCA_900109695
HaloferaxlarseniiJCM13917__GCA_000336955
HalorubrumspEb13__GCA_002252895
Halopigersalifodinae__GCA_900110455
NatrinemaspCBA1119__GCA_002572525
HaloarculavallismortisATCC29715__GCA_000337775
HaloferaxspSB3__GCA_001469875
HaloferaxprahovenseDSM18310__GCA_000336815
HaloferaxsulfurifontisATCCBAA-897__GCA_000337835
NatrialbamagadiiATCC43099__GCA_000025625
NatrialbamagadiiATCC43099__GCA_000337875
HalorubrumterrestreJCM10247__GCA_000337435
Haloarculavallismortis__GCA_900106715
HalorubrumdistributumJCM9100__GCA_000337055
HalorubrumdistributumJCM10118__GCA_000337335
HalorubrumspC191__GCA_002727095
Hrr_M52510
Hrr_M425108A
Hrr_ASP121
HalorubrumezzemoulenseGa2p__GCA_002252815
HalorubrumezzemoulenseLG1__GCA_002252735
HalorubrumezzemoulenseG37__GCA_002252835
HalorubrumezzemoulenseDSM17463__GCA_002114285
Hrr_SD690R
Hrr_12103MGM
HalorubrumezzemoulenseLD3__GCA_002252725
Halorubrumchaoviator__GCA_900188075
HalorubrumspSD612__GCA_002135005
HalorubrumezzemoulenseGa36__GCA_002252805
HalorubrumezzemoulenseEc15__GCA_002252875
HalorubrumspSD683__GCA_002135045
HalorubrumezzemoulenseFb21__GCA_002252865
HalorubrumkocuriiJCM14978__GCA_000337355
Halorubrumsaccharovorum__GCA_000700025
Hrr_SD626R
HalorubrumlacusprofundiATCC49239__GCA_000022205
Hrr_Sp7
Hrr_ASP200
HaloarculaspCBA1115__GCA_000827835
Natronomonasmoolapensis8811__GCA_000591055
Haloterrigenahispanica__GCA_900111485
Natronorubrumthiooxidans__GCA_900156475
NatrialbataiwanensisDSM12281__GCA_000337595
Halodesulfurarchaeumformicicum__GCA_001767315
NatronorubrumbangenseJCM10635__GCA_000337715
HalorubrumhochsteniumATCC700873__GCA_000337075
Halorubrumdistributum__GCA_002252935
HaloquadratumwalsbyiJ07HQW2__GCA_000415985
Halostagnicolakamekurae__GCA_900116205
HaloquadratumwalsbyiJ07HQW1__GCA_000415965
HalalkalicoccusjeotgaliB3__GCA_000337255
NatronococcusjeotgaliDSM18795__GCA_000337695
HalalkalicoccusjeotgaliB3__GCA_000196895
HalonotiusspJ07HN6__GCA_000416025
HaladaptatusspR4__GCA_001625445
HalostagnicolalarseniiXH-48__GCA_000517625
halophilicarchaeonJ07HX5__GCA_000415945
HalorubrumspJ07HR59__GCA_000416045
HalonotiusspJ07HN4__GCA_000416065
HaloferaxgibbonsiiATCC33959__GCA_000336775
HaloterrigenaturkmenicaDSM5511__GCA_000025325
HalostagnicolaspA56__GCA_000691505
Halalkalicoccuspaucihalophilus__GCA_001593955
Haloarchaeobiusiranensis__GCA_900103505
Halorubrumtropicale__GCA_001280455
HalorubrumcorienseDSM10284__GCA_000337035
NatronobacteriumgregoryiSP2__GCA_000337655
NatronobacteriumgregoryiSP2__GCA_000230715
Natronobacteriumgregoryi__GCA_900114025
Natrinemasalaciae__GCA_900110865
HalomicrobiummukohataeiDSM12286__GCA_000023965
Halococcushamelinensis100A6__GCA_000336675
Halomicrobiumzhouii__GCA_900114435
Halorubrumsodomense__GCA_900111935
Halopenitusmalekzadehii__GCA_900108505
Halanaeroarchaeumsulfurireducens__GCA_001011115
Halanaeroarchaeumsulfurireducens__GCA_001305655
HaloarculajaponicaDSM6131__GCA_000336635
NatrinemapellirubrumDSM15624__GCA_000337635
NatrinemapellirubrumDSM15624__GCA_000230735
HalococcusthailandensisJCM13552__GCA_000336715
HaloarculacaliforniaeATCC33799__GCA_000337755
HalopigerxanaduensisSH-6__GCA_000217715
Hrr_ASP1
HalorubrumspIb24__GCA_002252745
HalorubrumaidingenseJCM13560__GCA_000336995
HaloterrigenathermotoleransDSM11522__GCA_000337115
Natrinemaejinorense__GCA_002494345
Haloterrigenadaqingensis__GCA_001971705
Haloterrigenadaqingensis__GCA_900156445
HaloferaxelongansATCCBAA-1513__GCA_000336755
NatronorubrumsulfidifaciensJCM14089__GCA_000337735
HaloquadratumwalsbyiDSM16790__GCA_000009185
Halohastalitchfieldiae__GCA_900109065
Natronobacteriumtexcoconense__GCA_900104065
Halosimplexcarlsbadense2-9-1__GCA_000337455
HalorubrumspE3__GCA_002252955
Halopenituspersicus__GCA_900107205
HalorubrumspHd13__GCA_002252755
HalorubrumsaccharovorumDSM1137__GCA_000337915
HalorubrumpersicumC49__GCA_002727125
HaloarculaspK1__GCA_001647155
Halorientalispersicus__GCA_900110215
NatrialbaaegyptiaDSM13077__GCA_000337535
HalorubrumarcisJCM13916__GCA_000337015
HaloferaxmediterraneiATCC33500__GCA_000337295
Halobacteriumhubeiense__GCA_001488575
HaloferaxmediterraneiATCC33500__GCA_000685635
Natronolimnobiusbaerhuensis__GCA_002177135
HalobiformanitratireducensJCM10879__GCA_000337895
HaloferaxmediterraneiATCC33500__GCA_000306765
SalinarchaeumspHarcht-Bsk1__GCA_000403645
Halogranumrubrum__GCA_900114455
HaloarculahispanicaN601__GCA_000504565
HaloarculaargentinensisDSM12282__GCA_000336895
HaloarculahispanicaATCC33960__GCA_000223905
HalogeometricumpallidumJCM14848__GCA_000337095
NatrinemaspJ7-2__GCA_000281695
Halogeometricumlimi__GCA_900115785
HaloferaxdenitrificansATCC35960__GCA_000337795
NatronococcusamylolyticusDSM10524__GCA_000337675
NatrialbaspSSL1__GCA_001861355
NatrialbahulunbeirensisJCM10989__GCA_000337575
Halodesulfurarchaeumformicicum__GCA_001886955
HaloterrigenalimicolaJCM13563__GCA_000337475
NatronomonaspharaonisDSM2160__GCA_000026045
Halobiformahaloterrestris__GCA_900112205
Halopelagiuslongus__GCA_900100875
HaladaptatusspW1__GCA_001723155
HaloferaxmucosumATCCBAA-1512__GCA_000337815
Halorientalisregularis__GCA_900102305
halophilicarchaeonJ07HX64__GCA_000416085
halophilicarchaeonJ07HB67__GCA_000416105
Natronoarchaeumphilippinense__GCA_900215575
HalarchaeumacidiphilumMH1-52-1__GCA_000474235
NatrinemapallidumDSM3751__GCA_000337615
HalorubrumspEa1__GCA_002252965
HalorubrumlipolyticumDSM21995__GCA_000337375
NatrialbaasiaticaDSM12278__GCA_000337555
Halogranumamylolyticum__GCA_900110465
Haloterrigenasaccharevitans__GCA_001953745
HaloferaxspATCCBAA-646__GCA_000336855
HaloferaxspATCCBAA-645__GCA_000336835
HaloferaxspATCCBAA-644__GCA_000336975
Natronorubrumtexcoconense__GCA_900100335
Halogranumgelatinilyticum__GCA_900103715
Haloprofundusmarisrubri__GCA_001469955
Halopelagiusinordinatus__GCA_900113245
Halogeometricumrufum__GCA_900112175
HalogranumsalariumB-1__GCA_000283335
HalorubrumlitoreumJCM13561__GCA_000337395
HaloferaxspSB29__GCA_001469865
Halovenusaranensis__GCA_900100385
HalorientalisspIM1011__GCA_001989615
HalorubrumtebenquichenseDSM14210__GCA_000337415
HalorubrumspEa8__GCA_002252925
HalovivaxruberXH-70__GCA_000328525
NatrialbaceaearchaeonJWNM-HA15__GCA_002156705
HalobacteriumspDL1__GCA_000230955
HalococcussaccharolyticusDSM5350__GCA_000336915
Haloplanusvescus__GCA_900107665
HalococcussalifodinaeDSM8989__GCA_000336935
HalococcusmorrhuaeDSM1307__GCA_000336695
HaloarculaamylolyticaJCM13557__GCA_000336615
Haloferaxmassiliensis__GCA_001368915
HalorubrumspC3__GCA_002727105
HalorubrumspAJ67__GCA_000513435
Halobaculumgomorrense__GCA_900129775
HaloferaxalexandrinusJCM10717__GCA_000336735
HaloterrigenasalinaJCM13891__GCA_000337495
NatrialbachahannaoensisJCM10990__GCA_000337135
NatronolimnobiusinnermongolicusJCM12255__GCA_000337215
HalovivaxasiaticusJCM14624__GCA_000337515
NatrinemaversiformeJCM10478__GCA_000337195
HaloarculasinaiiensisATCC33800__GCA_000337275
CandidatusHalobonumtyrrellensisG22__GCA_000495475
NatrinemagariJCM14663__GCA_000337175
Hrr_Sp9
Hrr_11106MGM
Hrr_Sp3
Hrr_Sp5
HaloferaxvolcaniiDS2__GCA_000025685
HaloferaxvolcaniiDS2__GCA_000337315
Halobacteriumjilantaiense__GCA_900110535
HaloferaxspBAB2207__GCA_000328285
HalogeometricumborinquenseDSM11551__GCA_000172995
HalogeometricumborinquenseDSM11551__GCA_000337855
HaladaptatuspaucihalophilusDX253__GCA_000187225
HaladaptatuspaucihalophilusDX253__GCA_900142335
NatrinemaaltunenseJCM12890__GCA_000337155
HalorhabdusutahensisDSM12940__GCA_000023945
Natronorubrumsediminis__GCA_900108095
Halobellusclavatus__GCA_900107195
HalobacteriumsalinarumNRC-1__GCA_000006805
HalobacteriumsalinarumR1__GCA_000069025
Halolaminapelagica__GCA_900115675
HaloquadratumwalsbyiC23__GCA_000237865
HalolaminaspCBA1230__GCA_002025255
HalorubrumspWN019__GCA_002286985
Haloarcularubripromontorii__GCA_001280425
Halorubrumvacuolatum__GCA_900188065
HalorubrumhaloduransCb34__GCA_002252985
HalorhabdustiamateaSARL4B__GCA_000470655
HalorubrumcaliforniensisDSM19288__GCA_000336875
HalorhabdustiamateaSARL4B__GCA_000215915
Hrr_11GM103MGM
0.09

Figure S4. Maximum-likelihood phylogeny of cHG presence-absence matrix. An F81 model with empirical base-frequency was employed. The root was placed arbitrarily at the midpoint on account of the topology not bearing meaningful resemblance to the reference topology (cf. Figure 2).

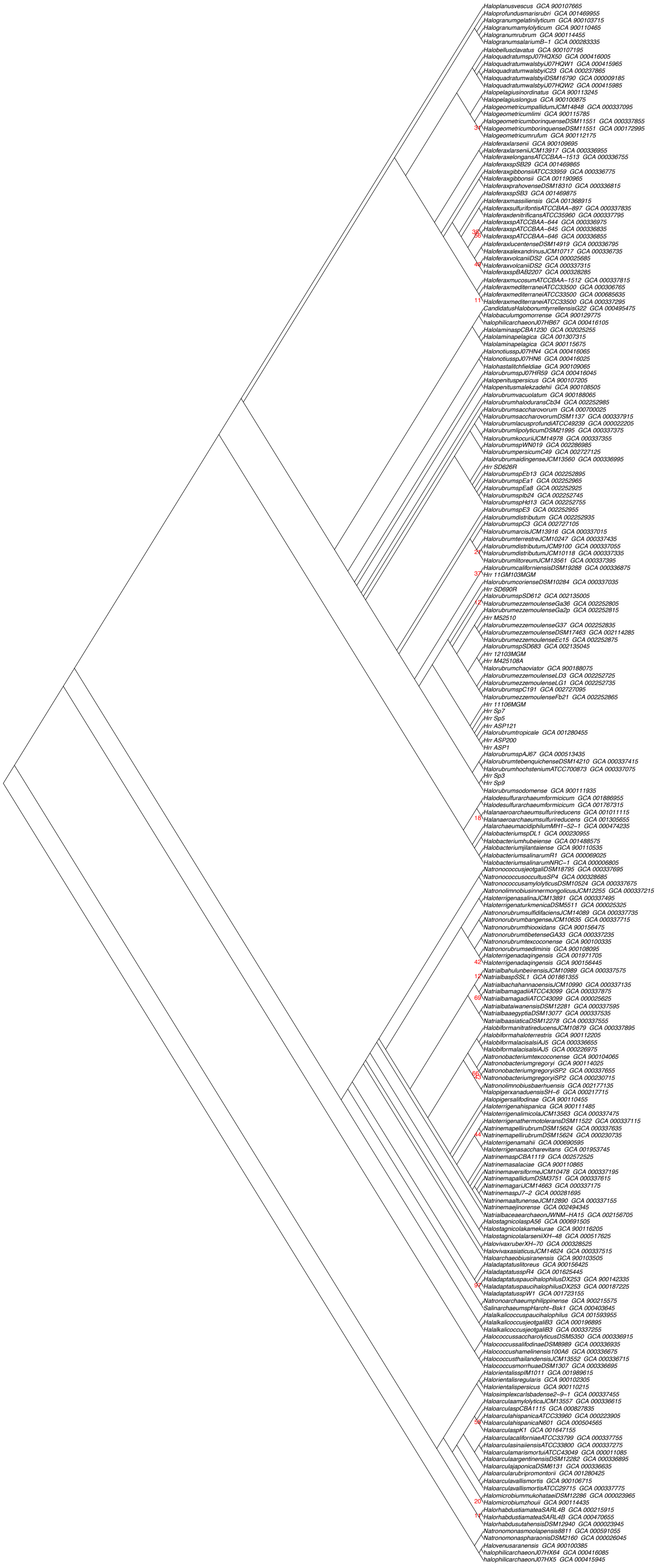

Haloplanusvescus GCA 900107665
Haloprofundusmarisrubri GCA 001469955
Halogranumgelatinilyticum GCA 900103715
Halogranumamylolyticum GCA 900110465
Halogranumrubrum GCA 900114455
HalogranumsalariumB−1 GCA 000283335
Halobellusclavatus GCA 900107195
HaloquadratumspJ07HQX50 GCA 000416005
HaloquadratumwalsbyiJ07HQW1 GCA 000415965
HaloquadratumwalsbyiC23 GCA 000237865
HaloquadratumwalsbyiDSM16790 GCA 000009185
HaloquadratumwalsbyiJ07HQW2 GCA 000415985
Halopelagiusinordinatus GCA 900113245
Halopelagiuslongus GCA 900100875
HalogeometricumpallidumJCM14848 GCA 000337095
Halogeometricumlimi GCA 900115785
HalogeometricumborinquenseDSM11551 GCA 000337855
HalogeometricumborinquenseDSM11551 GCA 000172995
Halogeometricumrufum GCA 900112175
Haloferaxlarsenii GCA 900109695
HaloferaxlarseniiJCM13917 GCA 000336955
HaloferaxelongansATCCBAA−1513 GCA 000336755
HaloferaxspSB29 GCA 001469865
HaloferaxgibbonsiiATCC33959 GCA 000336775
Haloferaxgibbonsii GCA 001190965
HaloferaxprahovenseDSM18310 GCA 000336815
HaloferaxspSB3 GCA 001469875
Haloferaxmassiliensis GCA 001368915
HaloferaxsulfurifontisATCCBAA−897 GCA 000337835
HaloferaxdenitrificansATCC35960 GCA 000337795
HaloferaxspATCCBAA−644 GCA 000336975
HaloferaxspATCCBAA−645 GCA 000336835
HaloferaxspATCCBAA−646 GCA 000336855
HaloferaxlucentenseDSM14919 GCA 000336795
HaloferaxalexandrinusJCM10717 GCA 000336735
HaloferaxvolcaniiDS2 GCA 000025685
HaloferaxvolcaniiDS2 GCA 000337315
HaloferaxspBAB2207 GCA 000328285
HaloferaxmucosumATCCBAA−1512 GCA 000337815
HaloferaxmediterraneiATCC33500 GCA 000306765
HaloferaxmediterraneiATCC33500 GCA 000685635
HaloferaxmediterraneiATCC33500 GCA 000337295
CandidatusHalobonumtyrrellensisG22 GCA 000495475
Halobaculumgomorrense GCA 900129775
halophilicarchaeonJ07HB67 GCA 000416105
HalolaminaspCBA1230 GCA 002025255
Halolaminapelagica GCA 001307315
Halolaminapelagica GCA 900115675
HalonotiusspJ07HN4 GCA 000416065
HalonotiusspJ07HN6 GCA 000416025
Halohastalitchfieldiae GCA 900109065
HalorubrumspJ07HR59 GCA 000416045
Halopenituspersicus GCA 900107205
Halopenitusmalekzadehii GCA 900108505
Halorubrumvacuolatum GCA 900188065
HalorubrumhaloduransCb34 GCA 002252985
Halorubrumsaccharovorum GCA 000700025
HalorubrumsaccharovorumDSM1137 GCA 000337915
HalorubrumlacusprofundiATCC49239 GCA 000022205
HalorubrumlipolyticumDSM21995 GCA 000337375
HalorubrumkocuriiJCM14978 GCA 000337355
HalorubrumspWN019 GCA 002286985
HalorubrumpersicumC49 GCA 002727125
HalorubrumaidingenseJCM13560 GCA 000336995
Hrr SD626R
HalorubrumspEb13 GCA 002252895
HalorubrumspEa1 GCA 002252965
HalorubrumspEa8 GCA 002252925
HalorubrumspIb24 GCA 002252745
HalorubrumspHd13 GCA 002252755
HalorubrumspE3 GCA 002252955
Halorubrumdistributum GCA 002252935
HalorubrumspC3 GCA 002727105
HalorubrumarcisJCM13916 GCA 000337015
HalorubrumterrestreJCM10247 GCA 000337435
HalorubrumdistributumJCM9100 GCA 000337055
HalorubrumdistributumJCM10118 GCA 000337335
HalorubrumlitoreumJCM13561 GCA 000337395
HalorubrumcaliforniensisDSM19288 GCA 000336875
Hrr 11GM103MGM
HalorubrumcorienseDSM10284 GCA 000337035
Hrr SD690R
HalorubrumspSD612 GCA 002135005
HalorubrumezzemoulenseGa36 GCA 002252805
HalorubrumezzemoulenseGa2p GCA 002252815
Hrr M52510
HalorubrumezzemoulenseG37 GCA 002252835
HalorubrumezzemoulenseDSM17463 GCA 002114285
HalorubrumezzemoulenseEc15 GCA 002252875
HalorubrumspSD683 GCA 002135045
Hrr 12103MGM
Hrr M425108A
Halorubrumchaoviator GCA 900188075
HalorubrumezzemoulenseLD3 GCA 002252725
HalorubrumezzemoulenseLG1 GCA 002252735
HalorubrumspC191 GCA 002727095
HalorubrumezzemoulenseFb21 GCA 002252865
Hrr 11106MGM
Hrr Sp7
Hrr Sp5
Hrr ASP121
Halorubrumtropicale GCA 001280455
Hrr ASP200
Hrr ASP1
HalorubrumspAJ67 GCA 000513435
HalorubrumtebenquichenseDSM14210 GCA 000337415
HalorubrumhochsteniumATCC700873 GCA 000337075
Hrr Sp3
Hrr Sp9
Halorubrumsodomense GCA 900111935
Halodesulfurarchaeumformicicum GCA 001886955
Halodesulfurarchaeumformicicum GCA 001767315
Halanaeroarchaeumsulfurireducens GCA 001011115
Halanaeroarchaeumsulfurireducens GCA 001305655
HalarchaeumacidiphilumMH1−52−1 GCA 000474235
HalobacteriumspDL1 GCA 000230955
Halobacteriumhubeiense GCA 001488575
Halobacteriumjilantaiense GCA 900110535
HalobacteriumsalinarumR1 GCA 000069025
HalobacteriumsalinarumNRC−1 GCA 000006805
NatronococcusjeotgaliDSM18795 GCA 000337695
NatronococcusoccultusSP4 GCA 000328685
NatronococcusamylolyticusDSM10524 GCA 000337675
NatronolimnobiusinnermongolicusJCM12255 GCA 000337215
HaloterrigenasalinaJCM13891 GCA 000337495
HaloterrigenaturkmenicaDSM5511 GCA 000025325
NatronorubrumsulfidifaciensJCM14089 GCA 000337735
NatronorubrumbangenseJCM10635 GCA 000337715
Natronorubrumthiooxidans GCA 900156475
NatronorubrumtibetenseGA33 GCA 000337235
Natronorubrumtexcoconense GCA 900100335
Natronorubrumsediminis GCA 900108095
Haloterrigenadaqingensis GCA 001971705
Haloterrigenadaqingensis GCA 900156445
NatrialbahulunbeirensisJCM10989 GCA 000337575
NatrialbaspSSL1 GCA 001861355
NatrialbachahannaoensisJCM10990 GCA 000337135
NatrialbamagadiiATCC43099 GCA 000337875
NatrialbamagadiiATCC43099 GCA 000025625
NatrialbataiwanensisDSM12281 GCA 000337595
NatrialbaaegyptiaDSM13077 GCA 000337535
NatrialbaasiaticaDSM12278 GCA 000337555
HalobiformanitratireducensJCM10879 GCA 000337895
Halobiformahaloterrestris GCA 900112205
HalobiformalacisalsiAJ5 GCA 000336655
HalobiformalacisalsiAJ5 GCA 000226975
Natronobacteriumtexcoconense GCA 900104065
Natronobacteriumgregoryi GCA 900114025
NatronobacteriumgregoryiSP2 GCA 000337655
NatronobacteriumgregoryiSP2 GCA 000230715
Natronolimnobiusbaerhuensis GCA 002177135
HalopigerxanaduensisSH−6 GCA 000217715
Halopigersalifodinae GCA 900110455
Haloterrigenahispanica GCA 900111485
HaloterrigenalimicolaJCM13563 GCA 000337475
HaloterrigenathermotoleransDSM11522 GCA 000337115
NatrinemapellirubrumDSM15624 GCA 000337635
NatrinemapellirubrumDSM15624 GCA 000230735
Haloterrigenamahii GCA 000690595
Haloterrigenasaccharevitans GCA 001953745
NatrinemaspCBA1119 GCA 002572525
Natrinemasalaciae GCA 900110865
NatrinemaversiformeJCM10478 GCA 000337195
NatrinemapallidumDSM3751 GCA 000337615
NatrinemagariJCM14663 GCA 000337175
NatrinemaspJ7−2 GCA 000281695
NatrinemaaltunenseJCM12890 GCA 000337155
Natrinemaejinorense GCA 002494345
NatrialbaceaearchaeonJWNM−HA15 GCA 002156705
HalostagnicolaspA56 GCA 000691505
Halostagnicolakamekurae GCA 900116205
HalostagnicolalarseniiXH−48 GCA 000517625
HalovivaxruberXH−70 GCA 000328525
HalovivaxasiaticusJCM14624 GCA 000337515
Haloarchaeobiusiranensis GCA 900103505
Haladaptatuslitoreus GCA 900156425
HaladaptatusspR4 GCA 001625445
HaladaptatuspaucihalophilusDX253 GCA 900142335
HaladaptatuspaucihalophilusDX253 GCA 000187225
HaladaptatusspW1 GCA 001723155
Natronoarchaeumphilippinense GCA 900215575
SalinarchaeumspHarcht−Bsk1 GCA 000403645
Halalkalicoccuspaucihalophilus GCA 001593955
HalalkalicoccusjeotgaliB3 GCA 000196895
HalalkalicoccusjeotgaliB3 GCA 000337255
HalococcussaccharolyticusDSM5350 GCA 000336915
HalococcussalifodinaeDSM8989 GCA 000336935
Halococcushamelinensis100A6 GCA 000336675
HalococcusthailandensisJCM13552 GCA 000336715
HalococcusmorrhuaeDSM1307 GCA 000336695
HaloorientalisspIM1011 GCA 001989615
Haloorientalisregularis GCA 900102305
Haloorientalispersicus GCA 900110215
Halosimplexcarlsbadense2−9−1 GCA 000337455
HaloarculaamylolyticaJCM13557 GCA 000336615
HaloarculaspCBA1115 GCA 000827835
HaloarculahispanicaATCC33960 GCA 000223905
HaloarculahispanicaN601 GCA 000504565
HaloarculaspK1 GCA 001647155
HaloarculacaliforniaeATCC33799 GCA 000337755
HaloarculasinaiiensisATCC33800 GCA 000337275
HaloarculamarismortuiATCC43049 GCA 000011085
HaloarculaargentinensisDSM12282 GCA 000336895
HaloarculajaponicaDSM6131 GCA 000336635
Haloarcularubripromontorii GCA 001280425
Haloarculavallismortis GCA 900106715
HaloarculavallismortisATCC29715 GCA 000337775
HalomicrobiummukohataeiDSM12286 GCA 000023965
Halomicrobiumzhouii GCA 900114435
HalorhabdustiamateaSARL4B GCA 000215915
HalorhabdustiamateaSARL4B GCA 000470655
HalorhabdusutahensisDSM12940 GCA 000023945
Natronomonasmoolapensis8811 GCA 000591055
NatronomonaspharaonisDSM2160 GCA 000026045
Halovenusaranensis GCA 900103385
halophilicarchaeonJ07HX64 GCA 000416085
halophilicarchaeonJ07HX5 GCA 000415945

Figure S5. Bootstrap support values of the presence-absence phylogeny mapped onto the ribosomal protein reference tree. All nodes with values of 10% or greater are shown. Only few groupings of organisms belonging to the same species or genus are moderately supported by both, the presence-absence phylogeny and the ribosomal proteins. The lack of mutual support between ribosomal reference tree and the RM system derived phylogeny confirms the discord between the datasets (ribosomal proteins and RM system related proteins).
